# Cuttlefish use the negation of dead prey to judge food value in foraging decision

**DOI:** 10.1098/rsbl.2026.0445

**Published:** 2026-10-21

**Authors:** Sophie Pei, Jun-Ya Lai, Chuan-Chin Chiao

**Affiliations:** ^1^ Kang Chiao International School, New Taipei City, Taiwan; ^2^ Department of Life Science, National Tsing Hua University, Hsinchu, Taiwan; ^3^ Institute of Systems Neuroscience, National Tsing Hua University, Hsinchu, Taiwan

**Keywords:** prey liveness, prey freshness, food quality, decision-making

## Abstract

Understanding how targeted prey conditions influence foraging decisions is a fundamental issue in animal behaviour. The present study investigated whether cuttlefish use prey liveness to assess overall food quality. Cuttlefish were allowed to choose between different levels of prey freshness and quantity. Experiments 1 and 2 examined cuttlefish choices between one live shrimp alone and one live shrimp paired with either one or four freshly dead shrimps. Experiment 3 evaluated their preferences of one live shrimp paired with either one freshly dead shrimp or one hourly dead shrimp. Experiment 4 assessed their choices between these two types of dead shrimp alone. Results showed that cuttlefish significantly avoided the side containing one live shrimp paired with one freshly dead shrimp, and this avoidance increased proportionally with the quantity of dead shrimp. However, cuttlefish choice did not differ significantly between freshly and hourly dead shrimps. These findings suggest that the presence of dead shrimp decreases a cuttlefish's perceived food value, and that motionless prey, regardless of their condition, serve as a negative indicator of overall food quality. This study demonstrates that cuttlefish assess total prey value based on the negation of dead prey, a mental process of intentionally removing hindrances during evaluation.

## 1. Introduction

How a predator assesses whether to target live prey (hunting) versus a dead carcass (scavenging) involves a sophisticated suite of cognitive processes, energetic calculations and behavioural trade-offs [1]. Facultative predators (animals that can both hunt and scavenge) must cognitively parse the health or state (live/dead) of a target species to maximize energetic returns in foraging decisions [2]. In some cases, animals also need to balance the pleasure value of food against the risk of injury [3]. Understanding how targeted prey condition influences foraging decisions is important in both behavioural ecology and decision neuroscience [4].

Cephalopods, which include cuttlefish, octopuses and squids, possess the most complex brains among invertebrates [5,6]. Cuttlefish are known for their sophisticated behaviours, ranging from episodic memory, future planning and delayed gratification [7–10]. Among these cognitive abilities, foraging behaviour and decision-making have attracted significant attention, as they offer insight into how cuttlefish evaluate various prey options based on previous experience and internal states [11,12]. In particular, previous research has shown that cuttlefish can distinguish different prey numbers and prefer large quantity in the food choice experiment, demonstrating their numeracy or number sense in foraging decisions [12].

Cuttlefish are opportunistic predators that eat a wide variety of prey when the opportunity arises [5]. However, they do not blindly strike at anything that moves; they execute a complex mental cost–benefit analysis before deciding to attack [6]. It is well known that a cuttlefish's decision to launch a visual attack is based on prey size and movement [13,14], and it is extremely difficult to rear cuttlefish on non-live diets, though some plasticity exists [15]. When given a choice between one live shrimp and two dead shrimp, cuttlefish actively abandoned the larger quantity of dead food in favour of the single live prey item [12]. This previous study clearly demonstrates that the value of dead prey is lower than that of live prey, and that cuttlefish foraging decisions are dependent on prey quality rather than quantity. However, it remains unknown whether the presence of dead prey would lower the perceived value of adjacent live prey.

In the present study, we investigated whether cuttlefish use overall prey liveness to assess perceived prey value. Specifically, freshly dead shrimp was used as a distractor in a two-alternative forced-choice (2AFC) test to examine the effect dead shrimp exerts on cuttlefish food choices. In addition, shrimps that had been dead for 1 hour were used in comparison with freshly dead shrimp to examine whether cuttlefish decisions depend on the freshness of dead prey. This study represents the first attempt to evaluate the cognitive capacity of cuttlefish to filter out irrelevant information in food choice and focus on the most valuable item during decision-making.

## 2. Material and methods

### (a) Animals and aquarium system

Juvenile pharaoh cuttlefish (*Sepia pharaonis*, now *Acanthosepion pharaonis*) were used in this study. The cuttlefish were hatched from eggs spawned by wild-caught females and reared by Aquatic Biotech Company (Yilan, Taiwan) between February and April of 2025. Following transport to National Tsing Hua University (Hsinchu, Taiwan), the animals were maintained in two 700 l closed recirculating aquaculture systems. Seawater was maintained at approximately 25°C with a salinity of 35 parts per thousand. The photoperiod was implemented in a 12/12-hour light/dark cycle. One month after hatching, juveniles were housed individually in porous floating containers. Container size was adjusted based on the animals' mantle length (ML): small containers (16 × 11 × 6 cm) were used for ML < 2 cm, and larger containers (24 × 16 × 6 cm) for ML > 2 cm. Before testing, cuttlefish were fed a diet of live freshwater shrimp (*Neocaridina denticulate*), one in each time and three times per day. The experiment was performed when animals reached two months of age, a timing when their average ML was approximately 2 cm. When individual animals died during the experiment, cuttlefish of the same age were introduced as replacements. In total, 26 juvenile cuttlefish were used in the present study.

### (b) Experimental setup and procedure

The experimental tank consisted of a large, transparent plastic testing arena filled with seawater. To minimize stress, a basket identical to the one used in the cuttlefish's home tanks was placed inside the arena to provide a familiar environment. An overhead digital video camera was mounted above the tank to record behavioural responses and choices. All experiments were conducted in the daytime and took place in a floating basket.

The experimental apparatus consisted of a two-chamber device made up of two small transparent plastic boxes (2.5 × 2.5 × 2.5 cm) separated by a protruding plastic sheet designed to force the cuttlefish to make an irrevocable choice (using a 2AFC design; figure 1). Depending on the experiment, different numbers of shrimps were placed in the two chambers, and the apparatus was lowered into the floating basket at the opposite side from the cuttlefish's location. To prevent startle behaviour when the experimental apparatus was lowered into the floating basket, cuttlefish were habituated to the presence of the apparatus and the lowering process before testing. In addition, cuttlefish were allowed a five-minute acclimation period before each trial began.

**Figure 1.**
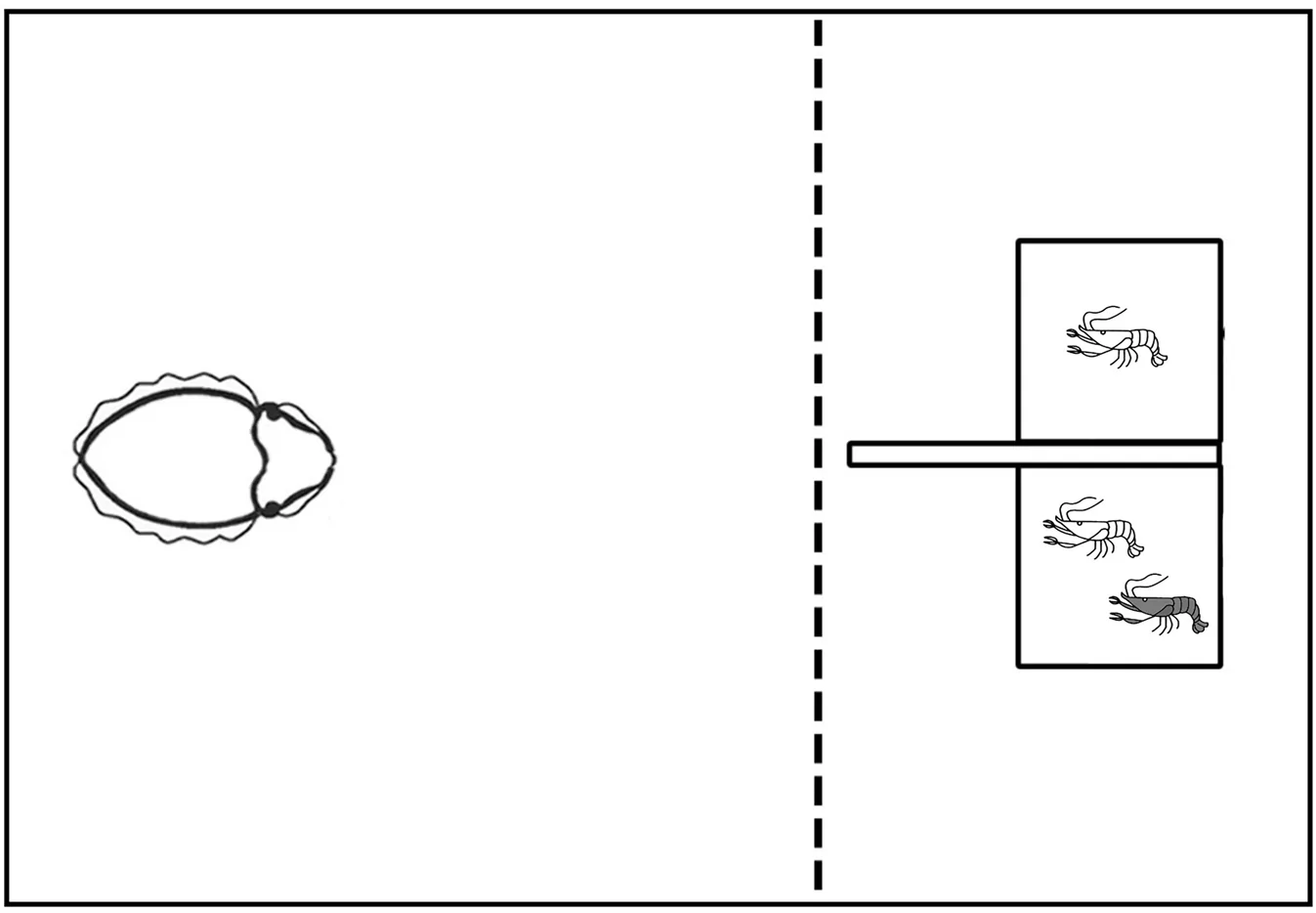
Schematic representation of the top view of the experimental setup. The two chambers containing shrimp are placed in front of the cuttlefish, and the animal is motivated to swim towards one of the two chambers. The imaginary dotted line indicates the decision point, where the cuttlefish determines the choice. In this example, the white and grey shrimp indicate live and freshly dead shrimp, respectively.

The experiment was initiated by allowing the cuttlefish to swim towards the two chambers and make a choice. Cuttlefish are active predators of live shrimp, which they capture by rapidly extending their two tentacles during a strike—a behaviour that is fundamentally visually driven in these animals [14]. To prevent passive avoidance learning (a known inhibition of predatory behaviour typically seen during ‘prawn-in-the-tube’ training) [16], the apparatus was lifted out of the water the moment the cuttlefish crossed a predetermined imaginary dotted line (figure 1). No food rewards were provided during any of the trials to prevent reinforcing the animal's decision-making process. Under no circumstances did a cuttlefish receive the shrimp inside the apparatus after making a choice. Unless specified otherwise, each cuttlefish underwent six tests per trial, across three distinct trials, culminating in 18 total tests per animal. To minimize the potential impact of visual lateralization, the left and right positions of the two chambers were sequentially swapped within each trial [17]. Once the animal completed its six tests within a given trial, it was fed an adequate amount of food in the absence of the apparatus. To guarantee that the shrimp remained vigorously active throughout testing, the contents of the chambers were refreshed every 10–15 minutes. Finally, the tops of the chambers were explicitly marked to indicate the type of shrimp contained within.

### (c) Experimental design

To investigate how prey quality and freshness influence foraging decisions, cuttlefish were subjected to four distinct experimental conditions. Each experiment included 9–13 animals. If a cuttlefish died during the experiment, a new animal was introduced as a replacement.

#### (i) Experiments 1 and 2 (effect of prey liveness)

In experiment 1, to evaluate whether dead shrimp provide a negative visual value in cuttlefish food choice, individual cuttlefish were offered a choice between one live shrimp versus a combination of one live and one freshly dead shrimp (L versus L + fD). This design aimed to examine whether the presence of a single dead shrimp has a negative impact on the perceived value of the adjacent live prey. In experiment 2, the quantity of dead shrimp was scaled up, offering a choice between one live shrimp versus a combination of one live and four freshly dead shrimps (L versus L + 4fD). This design aimed to examine whether the presence of multiple dead shrimp would further decrease the perceived prey liveness or slightly increase the perceived food value because of the larger quantity of prey.

#### (ii) Experiments 3 and 4 (effect of prey freshness)

In experiment 3, to assess the impact of prey freshness on cuttlefish food choice, individual cuttlefish were offered a choice between one live shrimp plus one freshly dead shrimp (dead in less than 30 minutes) versus one live shrimp alongside one shrimp that had been dead for at least one hour (L + fD versus L + hD). This design aimed to examine whether dead shrimp with various degrees of freshness affect cuttlefish foraging decisions, whereas a live prey was still present on both sides. In experiment 4, the live prey was removed, allowing cuttlefish to make a choice between one freshly dead shrimp versus 1-hour dead shrimp (fD versus hD). By removing the baseline of live shrimp, this design aimed to determine whether cuttlefish can distinguish the degree of prey freshness in the presence of dead shrimp alone. Because there was no live prey in experiment 4, cuttlefish were fasted before the trials over the previous night to encourage choice making; consequently, each animal was restricted to only 6 tests (instead of 18 tests in other experiments) for ethical and practical reasons.

### (d) Data analysis

The choices made by cuttlefish during 2AFC, expressed as a binary response, were analysed using a binomial generalized linear mixed model with a logit link function. The statistical analysis was implemented using the glmer function in the lme4 package in R. The model included overall choice preference as fixed effect, and random intercept for individual identity to account for repeated measurements in the data.

## 3. Results

In experiment 1, when presented with a choice between one live shrimp versus a combination of one live plus one freshly dead shrimp (L versus L + fD), cuttlefish significantly preferred the single live shrimp (*p* = 0.0106; figure 2A). The overall predicted probability of selecting the single live shrimp is 58.44%, which is significantly higher than the 50% chance baseline (odds ratio = 1.41). The estimated random intercept variance is less than 0.0001, indicating that between-subject variation is small and consistent with expected binomial sampling variance.

**Figure 2.**
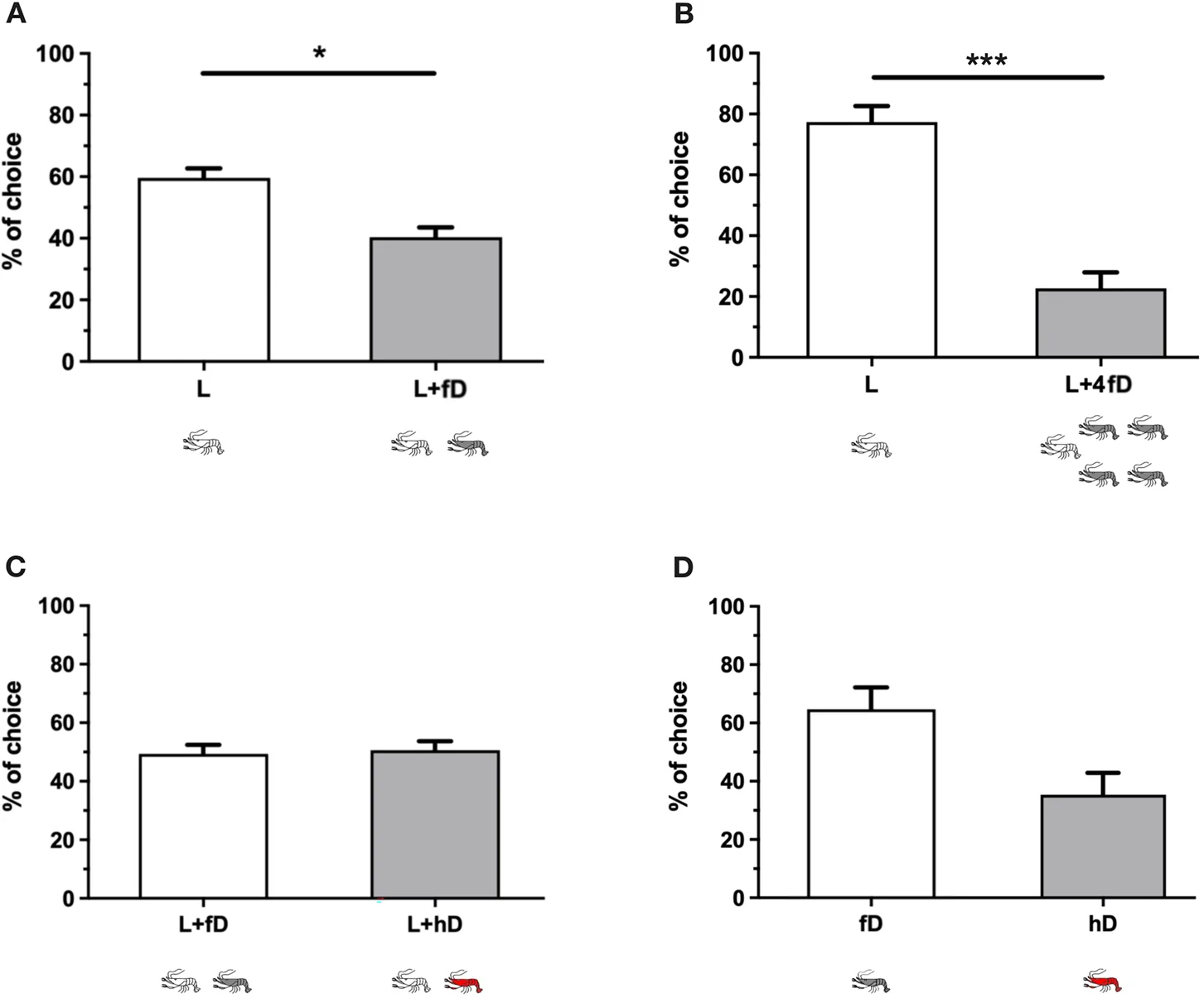
The presence of dead shrimp decreases cuttlefish's perceived food value, and motionless prey, regardless of their condition, is a negative indicator of overall food quality. The overall choices made by cuttlefish during 2AFC were represented as the percentage of choices. (A) Cuttlefish significantly preferred a single live shrimp (L) over a combination of one live and one freshly dead shrimp (L + fD) in experiment 1 (*n* = 13). (B) The preference for the single live shrimp was significantly greater when compared with the choice of one live shrimp paired with four freshly dead shrimp (L + 4fD) in experiment 2 (*n* = 9). (C) Cuttlefish did not show any preference when given a choice between one live plus one freshly dead shrimp (L + fD) versus one live plus one hourly dead shrimp (L + hD) in experiment 3 (*n* = 12). (D) In a choice between one freshly dead shrimp (fD) versus one hourly dead shrimp (hD) in experiment 4, cuttlefish had no statistically significant difference in their preying decision (*n* = 9). Error bars are s.e.m. *p < 0.05, ***p < 0.001.

This preference remained significant when the choice was between one live shrimp versus a group of one live and four freshly dead shrimps (L versus L + 4fD) in experiment 2 (*p* < 0.0001; figure 2B). Individuals exhibited an estimated average choice probability of 80.41% for the single live shrimp over the live plus four freshly dead shrimps, corresponding to an odds ratio of 4.10. The random intercept standard deviation is 0.4295, capturing modest between-subject variability across animals, whereas all individuals consistently showed preference above chance level. Notably, the preference for the one live shrimp was more robust when the quantity of dead prey (freshly dead shrimp) was increased from one to four in experiment 2 (i.e. the averaged percentage of choices for the single live shrimp increased from 59.6% in experiment 1 to 75.3% in experiment 2).

In experiment 3, when presented with a choice between one live shrimp coupled with one freshly dead shrimp versus one live shrimp coupled with one hourly dead shrimp (L + fD versus L + hD), cuttlefish showed no preference (*p* = 0.8913; figure 2C). Animals selected the live plus freshly dead shrimp at an estimated probability of 50.47%, which is relative to the 50% chance baseline. The estimated random intercept variance among individual subjects is less than 0.0001, indicating that between-individual differences do not exceed expected binomial sampling variation.

After the live prey was removed, given a choice between one freshly dead shrimp versus one hourly dead shrimp (fD versus hD) in experiment 4, cuttlefish did not show a significant preference either, though there was a trend towards preferring the freshly dead shrimp (*p* = 0.0567; figure 2D). It should be noted that the percentage of choices was calculated from only six tests per animal in experiment 4 (see §2 for details). However, the fixed effect intercept is positive (predicted probability = 65.48%, odds ratio = 1.90), indicating a preference towards freshly dead shrimp over hourly dead shrimp. Nevertheless, after accounting for individual variation, this difference approaches but remains just above the conventional two-tailed significance threshold. The estimated random intercept standard deviation across individuals is 0.4523, capturing moderate between-subject variation (preferences range from 33.33% to 100.00%, with 8 of 9 animals at or above 50%).

## 4. Discussion

The present study investigated how prey liveness and freshness influence foraging decisions in cuttlefish. By presenting live and dead shrimps together, we showed that cuttlefish not only innately reject dead prey, but the presence of dead prey can also decrease the perceived value of adjacent live prey. In addition, dead prey regardless of their freshness had no significant effect on the perceived quality of food. This study thus suggests that cuttlefish foraging decisions are dependent on overall prey quality, and that they evaluate prey value based on the perception and avoidance of dead prey.

Cuttlefish prefer one live shrimp over two dead shrimps when given a choice in a previous study [12], suggesting that the perceived value of dead prey is lower than that of live prey, and that their foraging decisions rely more on prey quality than prey quantity. In experiment 1 of the present study (L versus L + fD), it is apparent that the cuttlefish's perceived food value was significantly reduced by the presence of dead shrimp alongside a live shrimp (figure 2A). This indicates that dead prey are a disincentive in the cuttlefish's prey quality assessment. In experiment 2 (L versus L + 4fD), it is evident that cuttlefish further discounted the value of one live shrimp when there were four dead shrimps alongside (figure 2B). This indicates that a higher quantity of dead prey leads to a stronger negative influence on cuttlefish choices. This also implies that the presence of multiple dead prey items has an additive effect in reducing perceived prey quality.

It is well established that cuttlefish use polarization information to detect transparent prey [18]. Thus, cuttlefish may use the prey's polarization contrast, as well as locomotion, to distinguish live from dead shrimp. Although cuttlefish are colour-blind [19], they may discriminate between freshly dead and hourly dead shrimp based on other visual features, such as polarization and intensity contrasts. Note that there was no olfactory cue in this study, as all shrimp were kept in sealed plastic boxes during the experiment. The result in experiment 3 showed that cuttlefish exhibited no significant difference in choosing between one live shrimp plus one freshly dead shrimp versus one live shrimp plus one hourly dead shrimp (L + fD versus L + hD), suggesting that the value of live prey is much higher than that of dead prey regardless of the condition of the dead prey (figure 2C). However, when cuttlefish had to choose between one freshly dead shrimp versus one hourly dead shrimp (fD versus hD) in experiment 4, they showed a non-significant tendency favouring the freshly dead shrimp over the hourly dead shrimp (figure 2D). This result suggests that cuttlefish may evaluate both types of dead prey somewhat differently, and that freshly dead shrimp have a slightly higher value than hourly dead shrimp even though neither are their preferred food.

Cuttlefish are opportunistic predators, but they can switch to a selective foraging strategy when the availability of their preferred prey in the future is predictable [8]. This observation suggests that cuttlefish are capable of cognitively evaluating prey type and prey quality before making a foraging decision. In the present study, it is demonstrated that dead shrimp indeed have a negative value regarding prey quality, and cuttlefish may mentally calculate the overall value of food based on a negation process of dead shrimp. Although it is difficult to prove that cuttlefish are capable of subtraction operations, the fact that they can assess live shrimp value based on the presence or absence of dead shrimp (figure 2A–C) suggests that cuttlefish may estimate total prey liveness by subtracting the overall mass of dead prey from the mass of live ones. This is akin to the non-symbolic numerical abilities found in mammals, birds and honeybees [20,21], a cognitive capacity conserved in evolutionary distinct animal groups. Alternatively, instead of subtraction, cuttlefish may pay attention to the changing abundance of dead shrimp and use this information to assess the overall value of food.

Although it is well known that cuttlefish prefer live prey [14], it is less known why dead prey carries a negative value for them. The presence of dead prey might signal lower nutritional value, poor environmental conditions or danger. These could decrease appetite or increase stress in cuttlefish. It has been shown that stress induces a pessimistic judgement bias in cuttlefish [22]. Thus, the presence of dead prey could hinder their foraging decisions. In foraging ecology, differences in prey quality may be as important to predators as the costs of acquisition. For example, it has been shown that the quality of prey rather than the sheer quantity of food is a major determinant of foraging strategies employed by cetaceans to meet their specific energy requirements [23]. Similarly, cuttlefish weigh prey quality more than prey quantity when given a choice between one live shrimp versus two dead shrimps in a previous study [12]. In nature, cuttlefish must cognitively distinguish between live and dead prey to maximize their nutritional value and energetic return. The present study, showing that the presence of dead prey can significantly influence the assessment of prey quality, thus supports the premise that a value-based decision-making process is key to foraging success in cuttlefish.

## Supplementary Material



## Data Availability

All data are included in the supplementary material. Supplementary material is available online [24].
